# Clean air actions in China, PM_2.5_ exposure, and household medical expenditures: A quasi-experimental study

**DOI:** 10.1371/journal.pmed.1003480

**Published:** 2021-01-06

**Authors:** Tao Xue, Tong Zhu, Wei Peng, Tianjia Guan, Shiqiu Zhang, Yixuan Zheng, Guannan Geng, Qiang Zhang

**Affiliations:** 1 Institute of Reproductive and Child Health/Ministry of Health Key Laboratory of Reproductive Health and Department of Epidemiology and Biostatistics, School of Public Health, Peking University Health Science Center, Beijing, China; 2 Beijing Innovation Center for Engineering Science and Advanced Technology/State Key Joint Laboratory of Environment Simulation and Pollution Control, College of Environmental Science and Engineering, Peking University, Beijing, China; 3 School of International Affairs and Department of Civil and Environmental Engineering, Pennsylvania State University, University Park, State College, Pennsylvania, United States of America; 4 School of Public Health, Chinese Academy of Medical Sciences and Peking Union Medical College, Beijing, China; 5 College of Environmental Science and Engineering, Peking University, Beijing, China; 6 Center of Air Quality Simulation and System Analysis, Chinese Academy of Environmental Planning, Beijing, China; 7 School of Environment, Tsinghua University, Beijing, China; 8 Ministry of Education Key Laboratory for Earth System Modeling, Department of Earth System Science, Tsinghua University, Beijing China; Monash University, AUSTRALIA

## Abstract

**Background:**

Exposure to air pollution, a leading contributor to the global burden of disease, can cause economic losses. Driven by clean air policies, the air quality in China, one of the most polluted countries, has improved rapidly since 2013. This has enabled a unique, quasi-experiment to assess the economic impact of air pollution empirically.

**Methods and findings:**

Using a series of nation-scale longitudinal surveys in 2011, 2013, and 2015, we first examined the questionnaire-based medical expenditure changes before and after the policy intervention for air pollution. Using a state-of-the-art estimator of the historical concentration of particulate matters with diameter less than 2.5 μm (particulate matter (PM)_2.5_), we further quantified the association between household medical expenditure and PM_2.5_ using mixed-effect models of the repeated measurements from 26,511 households in 126 cities. Regression models suggest a robust linear association between reduced PM_2.5_ and saved medical expenditures, since the association did not vary significantly across models with different covariate adjustments, subregions, or subpopulations. Each 10 μg/m^3^ reduction in PM_2.5_ was associated with a saving of 251.6 (95% CI: 30.8, 472.3; *p*-value = 0.026) Yuan in per capita annual medical expenditure. However, due to limitations in data quality (e.g., self-reported expenditures), and imperfect control for unmeasured confounders or impact from concurrent healthcare reform in China, the causality underlying our findings should be further confirmed or refuted.

**Conclusion:**

In this study, we observed that compared with the PM_2.5_ reduction in 2013, the PM_2.5_ reduction in 2017 was associated with a saving of 552 (95% CI: 68, 1036) Yuan / (person × year), or approximately 736 billion Yuan (equivalent to 111 billion US dollar) per year nationally, which is equivalent to approximately 1% of the national gross domestic product of China.

## Abstract 2

In this observational study, Tao Xue and colleagues explore associations between medical expenses and changes to clean air policies in China since 2013.

## Author summary

**Why was this study done?:**

- Exposure to air pollution is a leading contributor to the global burden of disease.
- Mitigating air pollution can protect public health, so that can save medical expenditures on relevant diseases.
- The association between air pollution exposure and medical expenditures has been insufficiently studied.
- China conducted a series of clean air actions since 2013 and rapidly reduced the concentration of PM_2.5_, a major air pollutant.

**What did the researchers do and find?:**

- We examined medical expenditure changes before and after the policy intervention for air pollution, using a longitudinal nation-scale surveys in 2011, 2013, and 2015.
- After 2013, the PM_2.5_ concentration decreased more rapidly, but the medical expenditures increased more slowly.
- We also associated household medical expenditure to PM_2.5_ concentration using a mixed-effect models.
- We found that each 10 μg/m^3^ reduction in PM_2.5_ was associated with a saving of 251.6 Yuan in per capita annual medical expenditure.

**What do these findings mean?:**

- Air pollution was evidenced as a risk factor associated with increased medical expenditure.
- China’s clean air actions could bring a direct health benefit by saving medical expenditure.

## Introduction

Long-term exposure to ambient pollution has been identified as one of the leading causes of the global burden of disease [1], causing economic losses, including increased healthcare expenditures [2–6]. For instance, maternal exposure to particulate matter ≤2.5 μm in diameter (PM_2.5_) was estimated to contribute $760 million to medical costs in the United States of America, which is mainly spent on the treatment of PM_2.5_-associated preterm birth [4]. In 2015, the health burden of ambient PM_2.5_ and ozone resulted in a cost of $5.1 trillion in the 41 Organisation for Economic Co-operation and Development (OECD) countries [5]. Although the link between medical expenditures and air pollution is conceptually straightforward, real-world evidence of the association is rare.

Globally, China is one of the most polluted countries in terms of PM_2.5_. Long-term exposure to PM_2.5_ is estimated to cause approximately 1 million premature deaths annually [7]. To fight the severe air pollution, the central government adopted a series of stringent emission-control measures in 2013 [7,8]. These clean air actions included optimizing the industrial structure, improving end-of-pipe control, and reducing the residential use of unclean fuels [9]. Consequently, the national average PM_2.5_ concentration decreased from 67.4 μg/m^3^ in 2013 to 45.5 μg/m^3^ in 2017 [7]. Using an approach of atmospheric model, Zhang et al. evidenced these actions as the dominant contributions to the reduced concentration [10]. This intervention mitigated air pollution levels and hence the disease burden [7]. Therefore, it provides a quasi-experimental opportunity to study the impact of air pollution on health-related outcomes, including medical expenditures.

To examine the association between PM_2.5_ pollution and household medical expenditures, we conducted a quasi-experiment based on the China Health and Retirement Longitudinal Study (CHARLS) [11], which assessed the household expenditures of a representative sample of Chinese families in 2011, 2013, and 2015 using questionnaires. The self-reported household medical expenditures covered direct and indirect costs (e.g., healthcare-associated transportation payments). Based on comparisons between the CHARLS waves, we calculated the change in medical expenditures of each household before (Δ_before_ = expenditure_2013_ − expenditure_2011_) and after (Δ_after_ = expenditure_2015_ − expenditure_2013_) the clean air actions. If the assumed linkage between medical expenditures and PM_2.5_ exposure is true, the air quality improvement should cause a difference in the temporal change, i.e., Δ_after_ < Δ_before_. Using regression models, we quantified the association between state-of-the-art estimates of historical PM_2.5_ concentrations [12] and medical expenditures, after adjustment of various covariates. The analyses established an exposure–response function to evaluate the medical expenditures attributable to PM_2.5_ exposure. To check the robustness of our quasi-experimental study, we also analyzed the expenditures on clothing and recreation as negative controls.

## Methods

### Study households

**Fig 1:**
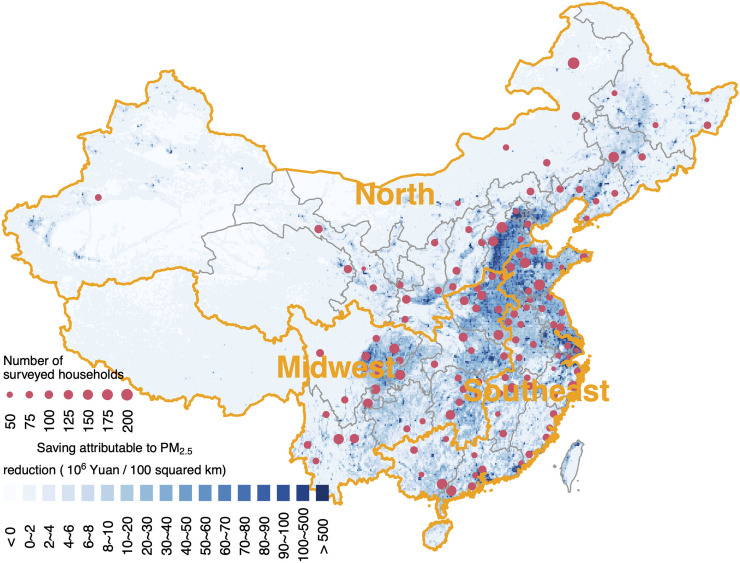
Locations of the surveyed households (red dots). The background colors show the annual savings in medical expenditures attributable to PM_2.5_, due to air quality improvement from 2013 to 2017. The geographic layers in the map were obtained from Natural Earth (http://www.naturalearthdata.com/). The places without available data (e.g., the South China sea islands) are not displayed in the maps. PM, particulate matter.

This study is based on the publically available CHARLS database [11], which is owned by the China Center for Economic Research, National School of Development, Peking University, and is accessed from the Peking University Open Research Data Platform (https://opendata.pku.edu.cn/) at no cost. The samples were the households visited by CHARLS, a nationwide longitudinal survey focusing on the health and socioeconomic status of the older Chinese population. The survey was designed as a cohort study, with a complex 4-stage sampling approach. The CHARLS interviewers have collected a representative sample of approximately 20,000 Chinese adults (Fig 1) every 2 years, since 2011. At least 1 adult member of each surveyed household was interviewed face to face by well-trained interviewers using a computer-assisted questionnaire. CHARLS also collected variables on demographic characteristics (e.g., education), behavioral risk factors (e.g., smoking), city of residence, and household characteristics (e.g., building type). The variables used in this study are listed in S1 Table. For details of the CHARLS study design, questionnaires, and survey processes, refer to the previous study [11]. CHARLS was approved by the Ethics Review Committee of the Peking University (IRB00001052–11015), and all participants signed the informed consent at the time of data collection.

This study was based on current data from the 2011, 2013, and 2015 CHARLS surveys. For longitudinal analysis, we focused on the households visited at least twice. A total of 26,099 households in 126 cities across China (Fig 1) were ultimately involved in the data analyses.

In CHARLS, the household expenditures for different purposes were measured using a standard questionnaire consistent across the different survey waves. If multiple family members were surveyed, the primary person who purchased food for the household was responsible for answering the questions about expenditures. The expenditures covered all of the household members who lived together and those who lived elsewhere for work or study but came home regularly. The money spent in the 12 months preceding the survey was counted. The number of household members was also collected by questionnaire in each wave. We utilized the per capita expenditures as dependent variables to account for the changes in family size. For household medical expenditures, the surveyed member was required to recall both the relevant direct and indirect costs. The indirect costs included, but were not limited to, payments for transportation to hospitals and payments for extra nutrition. Therefore, the variable reflected the out-of-pocket expenditures in household level, but was less indicative for the other types of healthcare costs (e.g., public medical expenditures). We also assessed clothing (defined as all payments for clothing and bedding) and recreation (defined as payments for purposes such as vacations) expenditures as negative controls.

### Environmental exposure

To evaluate the exposure to a nonoptimal ambient environment, we selected PM_2.5_ as a representative indicator of air pollutant in China. Because there was no nationwide monitoring network for PM_2.5_ before 2013 in China, historical PM_2.5_ concentrations were obtained from a state-of-the-art estimator, PM_2.5_ Hindcast Database for China (2000 to 2016) [12], which can be freely accessed at http://www.meicmodel.org/dataset-phd.html, or directly requested from its owners, the corresponding authors of this paper. For consistency, exposure assessments either before or after 2013 were based on the PM_2.5_ database. The historical estimator was generated from satellite measurements of aerosol and chemical transport model simulations, using a well-developed machine learning model [12]. The Hindcast PM_2.5_ concentrations were found to be in good agreement with the in situ observations on monthly (*R*^2^ = 0.71) and yearly (*R*^2^ = 0.77) scales and have been utilized in previous epidemiological studies [13]. The estimator was also evidenced to representative for the temporal trend in PM_2.5_ [12] and thus could be applied to study the impacts of air quality policies.

The PM_2.5_ data were generated for a 0.1° × 0.1° map of China by days. In the publically available CHARLS surveys, the households are located only at the city level to protect confidentiality. To match the environmental variable with the survey records spatially, we prepared gridded values of PM_2.5_ as city-level averages according to the prefecture map of China. Since the household medical expenditures mainly reflected the payments for medical services within 12 months before the survey (e.g., hospital visits, medical treatments, and inpatient care), this study focused on the concurrent ambient exposure associated with the diseases that might contribute to those costs (e.g., PM_2.5_-associated lower respiratory infections, and stroke). Therefore, we used the annual PM_2.5_ concentrations, averaged during the period covered by the surveyed medical expenditures, i.e., the 12 months preceding the survey month.

Climate variables, particularly ambient temperature, can also affect adult health, and may be a study confounder. To control for this, we used a series of gridded maps of temperature with a 0.1° × 0.1° resolution from our previous publication [13]. The temperature maps combined temperatures from (1) satellite remote sensing measurements, (2) a nationwide monitoring network, and (3) outputs from a weather forecast research model [13]. The temperature variables were fused using a universal kriging approach to improve the accuracy of the exposure assessment. The assembled temperature product was highly correlated with the monitored observations (*R*^2^ = 0.96) and was suitable for our study purpose. In the same way as we prepared the PM_2.5_ data, we calculated the annual and city-level averages of temperature as the variable used directly in this study.

### Statistical analyses

#### A discontinuity analysis

We conducted a discontinuity analysis to examine the causal effect of the clean air actions on medical expenditures. The analysis was similar to discontinuity regression [14], which has been used to explore the causal effects of air pollution on health. Discontinuity regression examines whether an air quality policy breaks the continuous trend (e.g., spatial or temporal pattern) in a health-related outcome (e.g., mortality or life expectancy). However, CHARLS screened only 3 cross sections of the population, which makes it difficult to construct a smoothed trend of medical expenditures for each household. Therefore, we applied a simplified version of discontinuity regression. We first derived the linear temporal trends in medical expenditures before (Δ_before_) and after (Δ_after_) the clean air actions for each household. Then, we compared the 2 linear trends within a household, using paired *t* tests. Since a significant difference between Δ_before_ and Δ_after_ indicates discontinuity in the temporal trend, our approach is similar to discontinuity regression and is called discontinuity analysis. Given the simplification, the approach is not as powerful as the discontinuity regression to examine a causal effect. In this study, the discontinuity analysis was applied at the household level, which increased its statistical power, compared with some ecological-level discontinuity regressions [14]. Because discontinuity analysis requires 3 cross sections of a study population, it could be applied only to the subset of 19,581 households that participated in all CHARLS waves in 2011, 2013, and 2015 (S1 Table).

#### Regression model

Difference-in-difference model was also commonly utilized to evaluate the causal effect based on the self-contrast before and after an intervention. A regression with fixed-effects has been known as a generalized version of the difference-in-difference model for the repeated measurements more than twice [15]. However, modeling fixed-effects (i.e., incorporating a dummy variable of household code into regression) can lead to the curse of dimensionality, which thus results in overfitting. Given that, random effects can be a more efficient approximation for the fixed-effects by reducing modeling complexity. For the main analyses, we associated the household medical expenditure per capita (*y*_*i*,*j*_) with the corresponding PM_2.5_ exposure using the following mixed-effects model:

$$y_{i,j}=\beta_{0}+\beta_{1}PM_{2.5,i,j}+f\left(T_{i,j}\right)+z_{i,j}\gamma +city_{i}+year_{i}+\eta \left(community_{i}\right)+\lambda \left(i\right),$$ (1)

where *i* and *j* are indexes for the household and CHARLS visit, respectively; *β*_0_ is the intercept; *β*_1_ is the coefficient measuring the effect of PM_2.5_; *f*(*T*_*i*,*j*_) is a spline function of temperature (*T*_*i*,*j*_) with 3 degrees of freedom; ***z***_*i*,*j*_ is a set of adjusted covariates; and ***γ*** is the corresponding regression coefficients. To adjust the city-level factors (e.g., economic growth rate) that affected medical expenditures but were unmeasured, we incorporated a fixed-effect term (*city*_*i*_); to adjust the long-term trend in expenditure, we involved a term of calendar year (*year*_*i*_); to control the correlation of the measured outcome within the same community or household, we used the random slope *η* or *λ*, respectively. The adjusted cofounders (***z***_*i*,*j*_) included (1) household characteristics (residence, child-rearing, care for parents, number of member (s) who eat together, and per capita wage); (2) indoor risk factors (indoor temperature, smoking or drinking and cooking energy type); (3) characteristics of the household head (marriage, education, sex, and age); (4) housing characteristics (building type, rent paid, in-house telephone, in-house internet, and household tidiness); and (5) health insurance coverages (not covered by any insurance or covered by insurance 1, 2, or 3; 1: urban employee basic medical insurance, 2: urban resident basic medical insurance, 3: rural new cooperative medical scheme). The regression model was applied to all samples; i.e., the 26,099 households with ≥2 visits. To test the association between PM_2.5_ and the negative controls, we replaced the medical expenditure per capita by the clothing or recreation expenditure per capita in the regression model. To make full use of the surveyed data, missing values for the above covariates (***z***_*i*,*j*_) were imputed based on the standard chained equation approach [16]. The approach draws imputations for a variable by fully conditional distributions on the rest variables and is advantageous when the multivariate joint distribution is complicated (e.g., our covariates included both continuous and categorical variables). Since the imputation could be potentially problematic, particularly for the covariate with a large fraction of missing values, we derived regressions with different sets of adjusted covariates. The model adjusted by less covariates was less influenced by the imputation procedure. To examine whether using the random terms was appropriate to model the household-specific effects, we also conducted parallel fixed-effects models [17].

#### Causal mediation analyses

To examine whether the estimated relationship between PM_2.5_ and medical expenditure was attributable to health, we conducted a mediation analyses. We assumed the relationship could be partially explained by the effect of PM_2.5_ on hospital admission, which has been evidenced by many extant studies. In the first stage of the mediation analysis, we first regressed an indicator for hospital admission with PM_2.5_ and other covariates, and the regression coefficient for PM_2.5_ was denoted by *α*; In the next stage, we regressed the medical expenditure with PM_2.5_, hospital admission, and the same set of covariates, and the coefficients for PM_2.5_ and hospital admission were denoted by *β*_direct_ and *γ*, respectively. Therefore, the total effect of PM_2.5_ on medical expenditure was quantified as *β*_total_ = *β*_direct_ + *β*_mediated_, where *β*_mediated_ denoted the effect attributable to the relationship between PM_2.5_ and hospital admission and was calculated as *β*_mediated_ = *αγ*. To test whether the estimated association between PM_2.5_ and medical expenditure was relied on the health effect of air pollution, we examined the null hypothesis of *β*_mediated_/*β*_total_ = 0. We utilized the mediation package for the statistical inference of mediation analyses [18].

#### Sensitivity analyses

We conducted several sensitivity analyses to examine the robustness of the estimated association between PM_2.5_ and household medical expenditures. First, we examined the between-subpopulation heterogeneity in the association between PM_2.5_ and medical expenditures. To test this, in the regression model (Eq 1), we added an interaction term between PM_2.5_ and a subgroup indicator, including residence, sex of household head, education of household head, marriage status of the household head, child-rearing, care for parents, building type, or rental payment. Second, we examined whether the association varied spatially. The analysis was based on a test of the interaction effect between PM_2.5_ and a regional indicator (southeast, midwest, and north, as shown in Fig 1). Third, we examined the curvature of the association using a nonlinear model, in which we replaced the linear term of PM_2.5_ with a penalized spline term. The optimal degree of the spline term was estimated using the regularization approach [19]. Fourth, to explore how the selection of samples changed the estimated association, we reevaluated the regression model (Eq 1) using 2 subsets of the 26,099 households. In 1 subset, we incorporated only CHARLS 2013 and 2015, which surveyed the households in the same season of the corresponding years. This subset model examines whether incorporating the baseline survey (CHARLS 2011, which is less comparable with CHARLS 2013 or 2015 in terms of screening time) changes the estimated association. In the other subset, we incorporated the 18,879 households that participated in all 3 CHARLS surveys. Consequently, the reevaluated regression model was based on the same dataset that was involved in the discontinuity analysis. Fifth, to explore how inflation affects our results, we calibrated the expenditures into monetary values at a constant 2010 price. In the analysis, we also tested whether different inflation–calibration methods would change the estimated association. Finally, using a bootstrap method [20], we also evaluated how the association estimator was affected by the exposure misalignment error caused by the usage of city-level PM_2.5_. For details of the method, please refer to S1 Text.

#### Nation-scale impact assessment

To evaluate how the clean air actions during 2013 to 2017 affected the household medical expenditures in China, we quantified the money saved attributable to the PM_2.5_ reduction (Δ*Y*) using the following equation:

$$\Delta Y_{t}=\Sigma_{s}\Delta y_{st}\times P_{s},\;\Delta y_{st}=\beta_{1}\times \left(PM_{2.5,s,t}-PM_{2.5,s,2013}\right),$$ (2)

where *s* and *t* denote the index for county and year, respectively; Δ*y*_*st*_ denotes the annual savings attributable to household medical expenditures per capita for the *s*-th county during the *t*-th year; *P*_*s*_ denotes the population size for the *s*-th county and was obtained from the nearest census in 2010; and *β*_1_ denotes the exposure–response relationship between PM_2.5_ and per capita medical expenditures. Assuming that our regression model was nationally representative, *β*_1_ could be obtained from the estimated coefficient (Table 1). Δ*Y*_*t*_ denotes the nation-scale sum of the savings attributable to the PM_2.5_ reduction in the *t*-th year (*t* = 2014, …, 2017). To increase the interpretability of the assessed impact, we compared the results with the reported healthcare expenditures from the China Statistics Yearbooks (http://www.stats.gov.cn/tjsj/ndsj/).

**Table 1:** Estimated associations between PM_2.5_ and household expenditures according to different models.

Model*	Subset^†^	Price adjustment^‡^	Expenditure per 10 μg/m^3^ increment in PM_2.5_		
			Medical	Clothing	Recreation
1	All samples	No adjustment	302.4 (87.2, 517.5)	−15.8 (−46.4, 14.8)	−24.3 (−87.7, 39.0)
2			265.7 (45.3, 486.2)	−13.0 (−44.4, 18.3)	−20.2 (−85.1, 44.6)
3			268.0 (47.3, 488.6)	−7.2 (−38.6, 24.2)	−11.2 (−76.2, 53.7)
4			249.5 (28.9, 470.2)	−9.4 (−40.9, 22.0)	−15.2 (−80.2, 49.8)
5			256.3 (35.7, 476.9)	−10.9 (−42.3, 20.5)	−17.5 (−82.5, 47.5)
6			251.6 (30.8, 472.3)	−17.1 (−48.5, 14.4)	−19.3 (−84.4, 45.8)
6	1		243.9 (21.3, 466.4)	−10.9 (−42.6, 20.7)	−13.9 (−79.6, 51.8)
6	2		246.6 (25.7, 467.5)	−20.3 (−51.7, 11.2)	−21.3 (−86.4, 43.9)
			Expenditure at 2010 price for 10 μg/m^3^ increment of PM_2.5_		
6	All samples	1	230.9 (36.9, 425.0)	−16.6 (−44.4, 11.3)	−17.0 (−75.8, 41.8)
6		2	237.7 (60.2, 415.2)	−14.4 (−40.3, 11.5)	−14.9 (−71.5, 41.7)
6		3	244.4 (49.6, 439.1)	−15.5 (−43.6, 12.6)	−16.6 (−76.4, 43.2)
6		4	258.9 (56.7, 461.0)	−14.5 (−43.6, 14.6)	−16.8 (−78.5, 44.9)

* Model 1: Unadjusted model. Model 2: Model 1 + nonlinear effect of temperature. Model 3: Model 2 + household characteristics (residence, child-rearing, parental care, number of member(s) who eat together, and per capita wage) + indoor risk factors (indoor temperature maintenance, smoking or drinking, cooking energy type, and heating energy type). Model 4: Model 3 + insurance coverages. Model 5: Model 4 + characteristics of the household head (marriage, education, sex, and age). Model 6: Model 5 + housing characteristics (building type, rent, in-house telephone, in-house internet, and household tidiness).

^†^ Subset 1: Households visited in 2013 and 2015. Subset 2: Households visited 3 times.

^‡^ Price adjustment 1: Adjusted ratios based on the gross domestic product. Price adjustment 2: Based on the gross domestic product of the service industry. Price adjustment 3: Based on the total consumer price index. Price adjustment 4: Based on the consumer price index of medical products and services.

Details of the adjusted ratios are given in S2 Table.

PM, particulate matter.

There was no prospective analysis plan; however, majority of analyses (discontinuity analyses, regression models, and impact assessments) were planned before the study. The mediator model was post hoc for explanatory purpose. When revising the manuscript, we further incorporated insurance variables into the regression models and derived the bootstrap analysis on the exposure measurement error. This study is reported as per the Strengthening the Reporting of Observational Studies in Epidemiology (STROBE) guideline (S1 Checklist).

## Results

From 126 cities (Fig 1), 26,511 households participated in CHARLS at least twice. Of them, 73.86% (19,581) were visited 3 times. The mean household medical expenditure was 1305 Yuan per capita per year, and the average PM_2.5_ exposure concentration was 55.2 μg/m^3^. S1 Table gives the detailed population characteristics of the samples and their PM_2.5_ exposures. Details on the spatiotemporal trends in PM_2.5_ are documented in S1 Fig. In most of populous areas of China, the PM_2.5_ concentrations were almost unchanged during 2011 to 2013, but significantly decreased during 2013 to 2015. The decreasing trends were more rapid in the hotspots of PM_2.5_ pollution (e.g., North China Plain and Sichuan Basin) compared with the rest of the areas.

**Fig 2:**
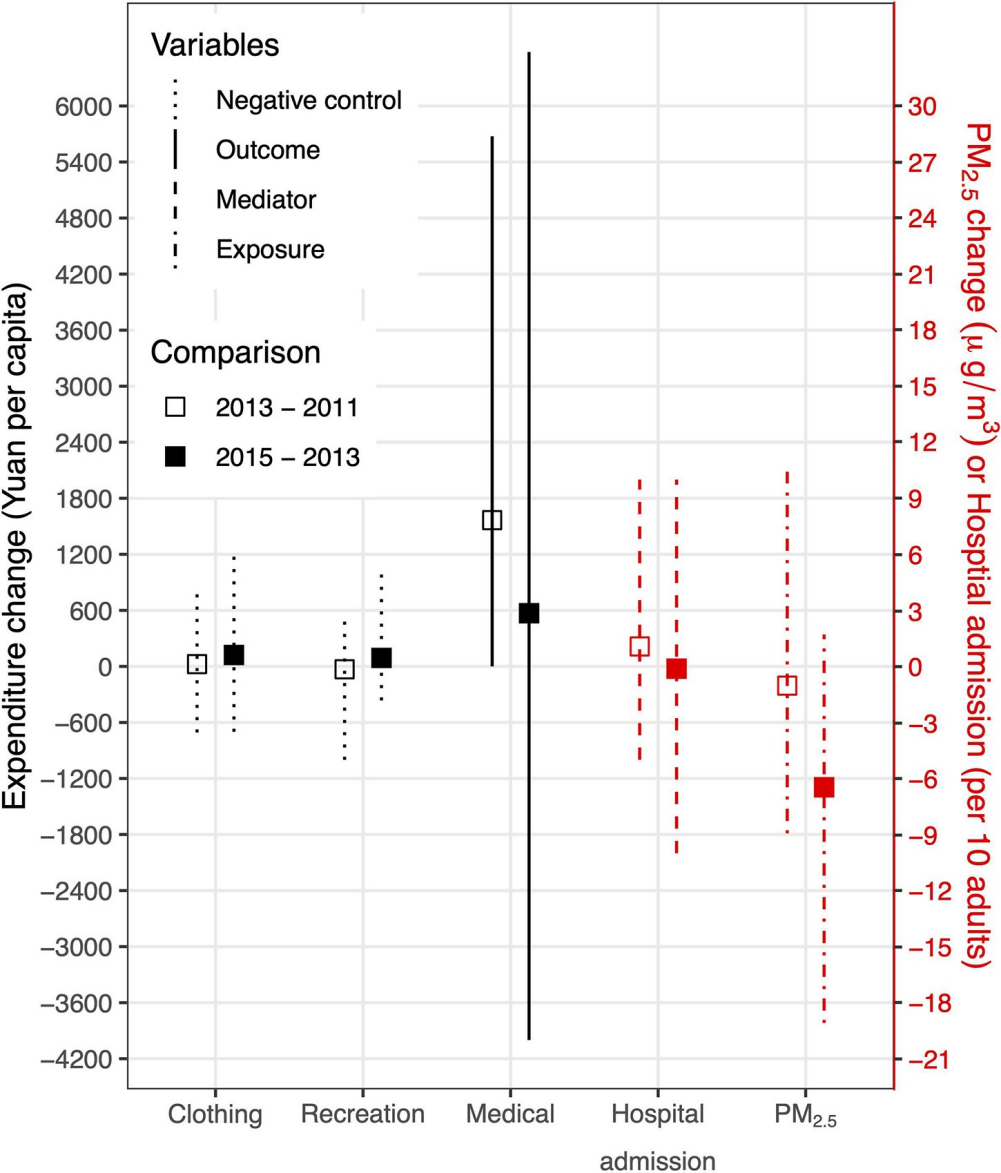
Comparisons of the annual expenditure change and corresponding PM_2.5_ change before (empty squares) and after (filled squares) the clean air actions in China. The error bars present the 95% confidence intervals for the changes, and the line types present different roles of the variables played in this study. PM, particulate matter.

A variety of socioeconomic parameters affect the changes in medical expenditures over time, such as aging, inflation, and the prices of medical services and medicine. However, the temporal change should be smooth and continuous with no intervention, in terms of population average. After the clean air actions, the mean Δ_after_ was 569 Yuan, which was much lower than the mean Δ_before_ (1,566 Yuan). The significant difference (*P*-value < 0.001; alternative hypothesis Δ_before_ > Δ_after_) suggests that the air quality policy correlated with a discontinuity in the temporal change in the medical expenditures (Fig 2). A similar difference in the temporal trend in PM_2.5_ confirmed the rapid improvement in air quality after 2013 (*P*-value < 0.001). By contrast, no significant discontinuity was found for the 2 negative controls, expenditures on clothing (*P*-value > 0.999) and recreation (*P*-value = 0.987). According to the discontinuity analysis, China’s clean air actions could cause the medical expenditure to decline.

Using the regression models for all 26,511 households, we examined the associations between the long-term PM_2.5_ exposure and medical expenditures with and without multiple adjustments (Table 1). The estimates using different models were statistically comparable. For instance, adjusting the covariates of insurance could partially control for the impact on medical expenditures from the healthcare policies that might concurrently change with the clean air actions. Incorporating insurance information into the model (i.e., changing from model 3 to 4, Table 1) slightly lowered the effect estimation, but the change was not statistically significant, given the uncertainties. Each 10 μg/m^3^ increment in PM_2.5_ was associated with a 251.6 Yuan (95% confidence interval [CI]: 30.2, 476.0) increment in the per capita medical expenditures in the fully adjusted model. By contrast, PM_2.5_ had no significant effect on the negative controls (clothing and recreation expenditures). We also found the results not sensitive to the choice of random- or fixed-effects, when modeling the household-specific variations in expenditure (S3 Table). Additionally, the mediation analyses evidenced that hospital admission was a significant mediator for the effect of PM_2.5_ on medical expenditure (S4 Table). Although the magnitude of the mediated effect was relatively small (which accounted for 17.2% [95% CI: 1.0%, 150.3%] of the total effect, according to the fully adjusted model), its significance was robust, given different models. By contrast, the significance level of the direct effect was sensitive to the choice of adjusted covariates. Those results suggest that the estimated economic effect of PM_2.5_ was relied on its adverse health effect.

**Fig 3:**
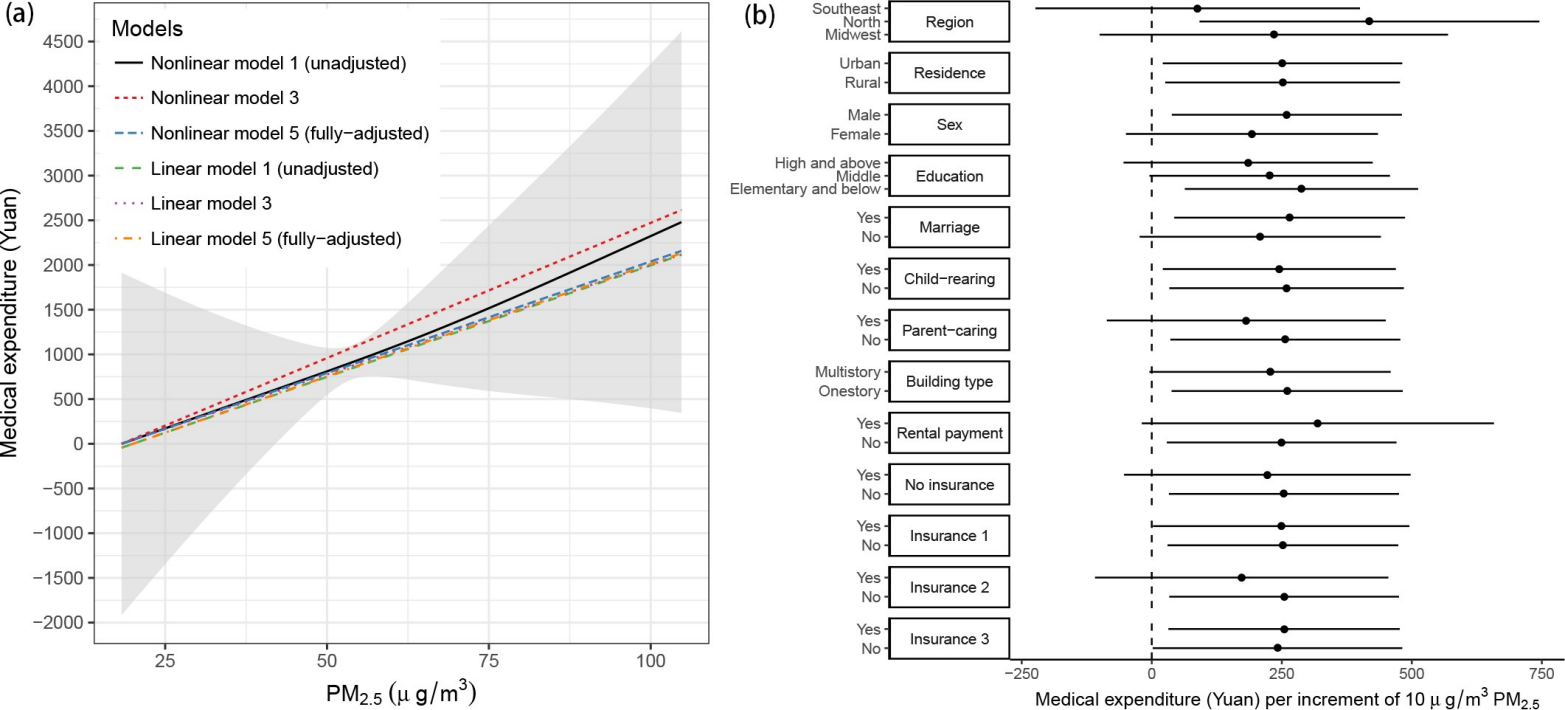
Estimated exposure–response functions between PM_2.5_ and medical expenditures (per capita) for different models (left) and subpopulations (right). The left panel shows the associations estimated using linear or nonlinear models (penalized spline models) with adjustments for different sets of covariates. The right panel shows the associations estimated from the fully adjusted linear models by different subgroups. Insurance 1: urban employee basic medical insurance, Insurance 2: urban resident basic medical insurance, Insurance 3: rural new cooperative medical scheme. PM, particulate matter.

In the sensitivity analyses of the robustness of the association (Fig 3), we first examined the linearity of the association between PM_2.5_ and medical expenditures. Penalized spline modeling of the nonlinearity confirmed that the medical expenditures increased linearly with PM_2.5_ exposure. Next, we examined the homogeneity of the associations between subpopulations. The subgroup had no significant modifying effect on the linkage. The association varied slightly between different subregions (*P*-value = 0.436); it was weaker in the southeast (coastal region) than in northern or midwest China. This might be caused by a higher fraction of toxic components in PM_2.5_ (e.g., carbonaceous particles emitted from fossil fuel combustion) in the north or midwest than that in the southeast [21]. The heterogeneity in the linkage between PM_2.5_ and medical expenditures is worthy of further study due to its implications for environmental policy. We also found comparable effects of PM_2.5_ between subgroups covered by different types of insurance, which acted as a surrogate for the progress in China’s health system reforms. The consistency suggests the effect of PM_2.5_ can be irrelevant with the reforms’ impact on medical expenditures. Additionally, adjusting the monetary values for various indicators, such as the consumer price index, can remove the impact of inflation on medical expenditures. We repeated our analyses using the adjusted expenditures (Table 1) and found that our results were not sensitive to inflation. Finally, using city-level PM_2.5_ could introduce the misalignment error in exposure assessment, which was showed to bring extra uncertainty embedded in the estimated association (S2 Fig). However, the estimated effect remains as significantly positive, after considering the misalignment error (S1 Text).

**Fig 4:**
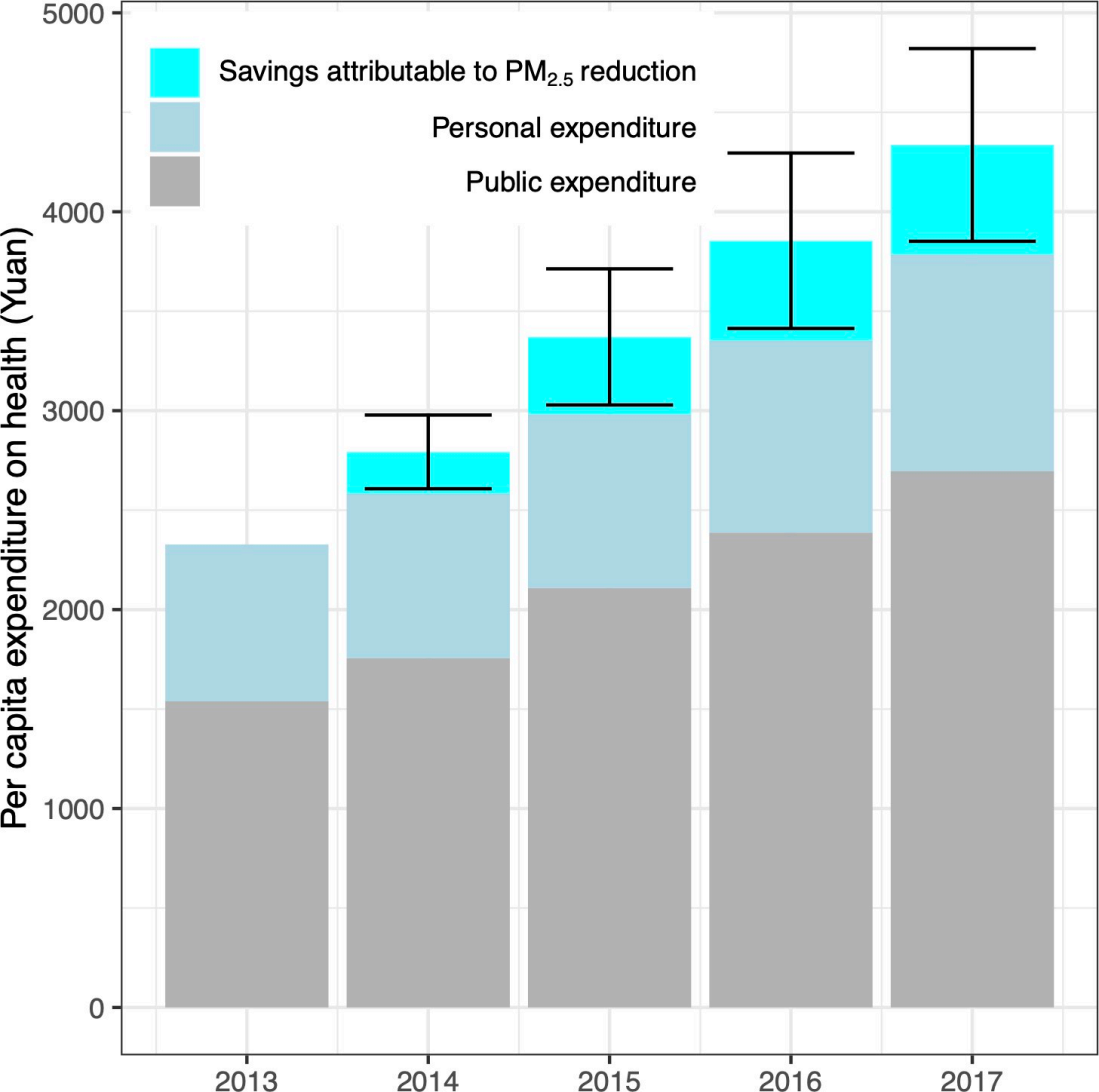
Estimated medical expenditure savings attributable to the PM_2.5_ reduction, in comparison with health expenditures in China from 2013 to 2017.

Using the established exposure–response function between PM_2.5_ and medical expenditures (the fully adjusted model, Table 1) and PM_2.5_ concentrations from 2013 to 2017 estimated from satellite measurements, in situ observations and emission inventories [7], we quantified the impact of China’s clean air actions on medical expenditures. Figs 1 and 4 show the spatial distribution and national summary, respectively. Due to the reduction in PM_2.5_ (from 67.4 μg/m^3^ in 2013 to 45.5 μg/m^3^ in 2017) [7], the per capita expenditure on medical services in 2017 was estimated to decrease by 552 Yuan (95% CI: 68, 1036) per year compared with the baseline value in 2013. The savings in medical expenses were comparable to the reported personal expenditures on healthcare (1,089 Yuan per capita) and accounts for 14.6% (95% CI: 1.8%, 27.4%) of the total medical expenditures (3,784 Yuan per capita). In total, the estimated savings on medical expenditures attributable to air quality improvement were 736 billion Yuan per year (95% CI: 91, 1381), equal to 0.9% (95% CI: 0.1%, 1.7%) of the gross domestic product (GDP) in 2017.

## Discussions

Based on a nationwide quasi-experimental study, we found that the policy-driven PM_2.5_ reduction in China was robustly associated with a saving in household medical expenditure. As far as we know, this study provides the first real-world evidence on beneficial economic impact from the clean air actions in China. Additionally, the quantitative association derived in this study between ambient PM_2.5_ concentration and medical expenditure can be a useful tool to assess the relevant economic impacts from air pollution.

To enable policymakers to quantify the potential benefits of air pollution control measures and conduct cost-benefit analyses to guide effective policy-making, it is important to assess the economic burden of air pollution [2]. Previous studies that estimated the economic impact of air pollution were knowledge-based modeling analyses [2,3,5,6]. The costs of air pollution were quantified indirectly, using approaches that included the cost of illness, revealed or stated preference approaches, or value of a statistical life. Our study is evidence-based. It goes beyond previous analyses by providing real-world evidence of the linkage between PM_2.5_ and household medical expenditures. A few cross-sectional studies have linked economic indicators and air pollution [22,23], but this approach cannot establish a credible causal relationship due to the potential failure to control confounders. Therefore, this quasi-experimental study added values by examining the linkage using causal inference models, which were capable to adjust confounders strictly.

The study also demonstrated that the clean air actions in China was associated with a significant reduction in the out-of-pocket expenditures on healthcare at national levels. During 2013 to 2017, the 32.5% reduction in PM_2.5_ concentrations saved 1% GDP by decreasing medical expenditures. Our estimates are comparable in scale with previous results. For instance, Yang and colleagues estimated that the 10.8% reduction in PM_2.5_ during 2015 to 2020 was associated with a 0.64% (95% CI: 0.24%, 1.04%) savings in medical expenditures [23]. However, our assessment that focused on household medical expenditures, ignored the effects of PM_2.5_ on public healthcare expenditures [24], relevant welfare losses, and the loss of lives. Therefore, the underlying economic benefits attributable to the clean air actions could be much larger than our estimates.

We recognize that this study has potential limitations. First, the expenditure variables in CHARLS are questionnaire-based [11] and thus can introduce recall bias into this study. The data quality and interindividual incomparability of the indicators can lead to high uncertainty. Although our analyses relied on within-household comparisons, which could minimize the influence of the inconsistency, errors in the expenditure variables might cause misclassifications in the outcome and thus bias the estimated associations. Second, due to the lack of air quality monitoring data in China before 2013, the exposure assessments were based on estimates of historical PM_2.5_. Although the estimates are in good agreement with in situ observations, particularly at monthly and yearly scales [12], the uncertainties in PM_2.5_ assessments might introduce exposure misclassification (particularly before 2013), which could bias our results. Usually, the random errors in the exposure assessment might lead to an underestimated association and an underestimated uncertainty embedded in the association (S2 Fig). Similarly, exposure misclassifications might also arise from measurement errors due to the usage of city-level PM_2.5_. More detailed analyses on this limitation are documented in the S1 Text. Third, due to the lack of details on the medical expenditures in the CHARLS surveys, we were unable to derive the disease-specific exposure–response functions for the economic effects of PM_2.5_, which limited the interpretability of our findings. Fourth, although we applied multiple causal inference models (including discontinuity analysis, a modified version of difference-in-difference regression, and a mediator analysis), the analyses were not perfect due to multiple reasons. For instance, due to the lack of long-term follow-up data, we could only examine the discontinuity in expenditures using a simplified method rather than the standard method of regression with discontinuity, which lowered the capacity of causal inference. Fifth, although the association between PM_2.5_ and medical expenditures was estimated as homogenous, its nationwide representativeness and generalizability should be questioned. When applying the estimated association to evaluate the economic loss attributable to PM_2.5_ exposure, differences between the target population and CHARLS can lead to a biased result. Further usage of our findings should be cautious. Given these limitations, this study should act as the first investigation on the nexus of air pollution, disease, and economics, and the causality underlying our findings should be confirmed or refuted by future studies.

## Conclusions

Our findings not only enrich evidences on the economic losses due to health effects of air pollution exposure, but also show that the PM_2.5_ reduction was associated with a considerable saving of household medical expenditure, after implementing the clean air actions in China.

## Supporting information

S1 Checklist STROBE Statement—checklist of items that should be included in reports of observational studies. (DOCX)

S1 Table Characteristics of the study population. (DOCX)

S2 Table Ratios used to adjust medical expenditures to the values at constant 2010 prices. (DOCX)

S3 Table Estimated associations between PM_2.5_ and household expenditures by fixed-effects models with different sets of adjusted covariates. (DOCX)

S4 Table Results of causal mediation models adjusted by different sets of covariates. (DOCX)

S1 Fig Spatiotemporal trends in PM_2.5_ before (2011 to 2013) and during the clean air actions (2013 to 2015). In each pixel, the trend is quantified using a least-squared regression of monthly PM_2.5_ concentrations against the temporal index. The geographic maps were obtained from Natural Earth (http://www.naturalearthdata.com/). The places without available data (e.g., the South China sea islands) are not displayed in the maps. PM, particulate matter.
(DOCX)

S2 Fig Results from the bootstrap simulations for errors attributable to the exposure misalignments, due to the usage of city-level PM_2.5_. PM, particulate matter. (DOCX)

S1 Text A bootstrap method to assess exposure misalignment errors. (DOCX)

## Abbreviations

CHARLS: China Health and Retirement Longitudinal Study
CI: confidence interval
GDP: gross domestic product
OECD: Organisation for Economic Co-operation and Development
PM: particulate matter
STROBE: Strengthening the Reporting of Observational Studies in Epidemiology
