# Supplementary material for: Clean air actions in China, PM2.5 exposure, and household medical expenditures: A quasi-experimental study
Source: PLoS Med. 2021 Jan 6;18(1):e1003480. doi: 10.1371/journal.pmed.1003480 (PMC7787388; doi:10.1371/journal.pmed.1003480)
Supplement: S1 Table — (DOCX) [file pmed.1003480.s002.docx]

S1 Table. Characteristics of the study population.

| Variable | Value | Summary |
| --- | --- | --- |
| Categorical variables | | N (Percentage of total visit-households) |
| Total | | 26511 (100.00%) |
| Visit number | 2 | 6930 (26.14%) |
|  | 3 | 19581 (73.86%) |
| Subregion | Midwest | 8970 (33.84%) |
|  | North | 7453 (28.11%) |
|  | Southeast | 10088 (38.05%) |
| Residence | Rural | 16474 (62.14%) |
|  | Urban | 10037 (37.86%) |
| Child-rearing | No | 12752 (48.10%) |
|  | Yes | 13755 (51.88%) |
|  | Unknown | 4 (0.02%) |
| Care of parents | No | 24940 (94.07%) |
|  | Yes | 1567 (5.91%) |
|  | Unknown | 4 (0.02%) |
| Number of member(s) who eat together | < 1 | 3424 (12.92%) |
|  | 2 | 8880 (33.50%) |
|  | 3 | 4980 (18.78%) |
|  | 4 | 3541 (13.36%) |
|  | 5 | 3057 (11.53%) |
|  | 6+ | 2608 (9.84%) |
|  | Unknown | 21 (0.08%) |
| Indoor temperature | Very hot | 275 (1.04%) |
|  | Hot | 2341 (8.83%) |
|  | Bearable | 22239 (83.89%) |
|  | Cold | 900 (3.39%) |
|  | Very cold | 82 (0.31%) |
|  | Unknown | 674 (2.54%) |
| Smoking or drinking | No | 12644 (47.69%) |
|  | Yes | 13275 (50.07%) |
|  | Unknown | 592 (2.23%) |
| Cooking energy type | Clean | 13620 (51.37%) |
|  | Unclean | 12624 (47.62%) |
|  | Unknown | 267 (1.01%) |
| Marriage | Cohabitated | 16 (0.06%) |
|  | Divorced | 337 (1.27%) |
|  | Married | 19741 (74.46%) |
|  | Married, living separately | 1625 (6.13%) |
|  | Never | 333 (1.26%) |
|  | Living separately | 128 (0.48%) |
|  | Windowed | 4315 (16.28%) |
|  | Unknown | 16 (0.06%) |
| Education | Elementary | 14464 (54.56%) |
|  | Middle | 3513 (13.25%) |
|  | High and above | 5838 (22.02%) |
|  | Unknown | 2696 (10.17%) |
| Sex | Female | 4029 (15.20%) |
|  | Male | 22477 (84.78%) |
|  | Unknown | 5 (0.02%) |
| Building type | One-story | 15334 (57.84%) |
|  | Multi-story | 11112 (41.91%) |
|  | Unknown | 65 (0.25%) |
| Rent | No | 15334 (57.84%) |
|  | Yes | 11112 (41.91%) |
|  | Unknown | 65 (0.25%) |
| In-house telephone | No | 16443 (62.02%) |
|  | Yes | 10047 (37.90%) |
|  | Unknown | 21 (0.08%) |
| In-house internet | No | 20838 (78.60%) |
|  | Yes | 5590 (21.09%) |
|  | Unknown | 83 (0.31%) |
| Household tidiness | Excellent | 2358 (8.89%) |
|  | Very clear | 5597 (21.11%) |
|  | Clear | 9088 (34.28%) |
|  | Fair | 7041 (26.56%) |
|  | Poor | 1764 (6.65%) |
|  | Unknown | 663 (2.50%) |
| Not covered by any health insurance | Yes | 1358 (5.12%) |
|  | No | 24754 (93.37%) |
|  | Unknown | 399 (1.51%) |
| Covered by insurance 1: urban employee basic medical insurance^#^ | Yes | 22321 (84.2%) |
|  | No | 4128 (15.57%) |
|  | Unknown | 62 (0.23%) |
| Covered by insurance 2: urban resident basic medical insurance^#^ | Yes | 24543 (92.58%) |
|  | No | 1901 (7.17%) |
|  | Unknown | 67 (0.25%) |
| Covered by insurance 3: rural new cooperative medical scheme^#^ | Yes | 5621 (21.2%) |
|  | No | 20858 (78.68%) |
|  | Unknown | 32 (0.12%) |
| Continuous variables | | Mean (SD, 90% CI)* |
| Per capita medical expenditure (Yuan) | | 1305 (6261, 0~5000) |
| Per capita clothing expenditure (Yuan) | | 422 (993, 0~1500) |
| Per capita recreation expenditure (Yuan) | | 275 (2073, 0~1200) |
| Hospital admission (Person-time per adult) | | 0.2 (0.5, 0.0~1.0) |
| Long-term exposure to PM_2.5_ (μg/m^3^) | | 55.2 (18.8, 26.7~88.5) |
| Temperature (°C) | | 24.3 (5.0, 14.7~29.4) |
| Per capita wage (Yuan) | | 3616 (8761, 0~17327) |

* SD: standard deviation; CI: confidence interval.

# A household can be covered by multiple types of health insurance.
