# Supplementary material for: Clean air actions in China, PM2.5 exposure, and household medical expenditures: A quasi-experimental study
Source: PLoS Med. 2021 Jan 6;18(1):e1003480. doi: 10.1371/journal.pmed.1003480 (PMC7787388; doi:10.1371/journal.pmed.1003480)
Supplement: S2 Table — (DOCX) [file pmed.1003480.s003.docx]

S2 Table. Ratios used to adjust medical expenditures to the values at constant 2010 prices.

Price adjustment 1

| Year | GDP at constant 2010 prices (100 million Yuan) | GDP (100 million Yuan) | Adjusted ratio |
| --- | --- | --- | --- |
| 2010 | 413,030.3 | 413,030.3 | 1.00 |
| 2011 | 452,429.9 | 489,300.6 | 0.92 |
| 2012 | 487,976.2 | 540,367.4 | 0.90 |
| 2013 | 525,835.4 | 595,244.4 | 0.88 |
| 2014 | 564,194.4 | 643,974 | 0.88 |
| 2015 | 603,124.9 | 689,052.1 | 0.88 |

GDP: gross domestic product. GDP values were obtained from the China Statistics Yearbook 2018 (http://www.stats.gov.cn/tjsj/ndsj/2018/indexch.htm).

Price adjustment 2

| Year | Tertiary industry GDP at constant 2010 prices (100 million Yuan) | Tertiary industry GDP (100 million Yuan) | Adjusted ratio |
| --- | --- | --- | --- |
| 2010 | 182,038.0 | 182,038.0 | 1.00 |
| 2011 | 199,310.5 | 216,098.6 | 0.92 |
| 2012 | 215,283.7 | 244,821.9 | 0.88 |
| 2013 | 233,150.5 | 277,959.3 | 0.84 |
| 2014 | 251,352.9 | 308,058.6 | 0.82 |
| 2015 | 271,954.1 | 346,149.7 | 0.79 |

The tertiary industry GDP values were obtained from the China Statistics Yearbook 2018 (http://www.stats.gov.cn/tjsj/ndsj/2018/indexch.htm).

Price adjustment 3

| Year | CPI (%) | Adjusted ratio |
| --- | --- | --- |
| 2010 | 103.3 | 1.00 |
| 2011 | 105.4 | 0.95 |
| 2012 | 102.6 | 0.92 |
| 2013 | 102.6 | 0.90 |
| 2014 | 102 | 0.88 |
| 2015 | 101.4 | 0.87 |

CPI: consumer price index. CPI values were obtained from the National Bureau of Statistics of China (http://data.stats.gov.cn/).

Price adjustment 4

| Year | CPI of medical services and products (%) | Adjusted ratio |
| --- | --- | --- |
| 2010 | 103.3 | 1.00 |
| 2011 | 102.9 | 0.97 |
| 2012 | 101.7 | 0.96 |
| 2013 | 101.5 | 0.94 |
| 2014 | 101.7 | 0.93 |
| 2015 | 102.7 | 0.90 |

Values of CPI of medical service and product were obtained from the National Bureau of Statistics of China (http://data.stats.gov.cn/).
