# Supplementary material for: Clean air actions in China, PM2.5 exposure, and household medical expenditures: A quasi-experimental study
Source: PLoS Med. 2021 Jan 6;18(1):e1003480. doi: 10.1371/journal.pmed.1003480 (PMC7787388; doi:10.1371/journal.pmed.1003480)
Supplement: S3 Table — (DOCX) [file pmed.1003480.s004.docx]

S3 Table Estimated associations between PM_2.5_ and household expenditures by fixed-effects models with different sets of adjusted covariates.

| Model^*^ | Expenditure per 10 μg/m^3^ increment in PM_2.5_ | | |
| --- | --- | --- | --- |
|  | Medical | Clothing | Recreation |
| 1 | 300.1 (82.6, 517.6) | -10.1 (-40.9, 20.7) | -21.0 (-84.5, 42.5) |
| 2 | 256.7 (34.2, 479.2) | -5.3 (-36.9, 26.2) | -15.0 (-80.0, 50.0) |
| 3 | 265.1 (42.3, 487.9) | -4.5 (-36.0, 27.0) | -12.3 (-77.4, 52.7) |
| 4 | 249.5 (26.5, 472.4) | -4.8 (-36.3, 26.7) | -11.9 (-77.0, 53.2) |
| 5 | 260.8 (38.1, 483.5) | -4.7 (-36.2, 26.9) | -13.5 (-78.6, 51.7) |
| 6 | 253.1 (30.2, 476.0) | -6.7 (-38.2, 24.9) | -12.4 (-77.6, 52.8) |

* Model 1: Unadjusted model;
Model 2: Model 1 + nonlinear effect of temperature;
Model 3: Model 2 + household characteristics (residence, child-rearing, parental care, number of member(s) who eat together, and per capita wage) + indoor risk factors (indoor temperature maintenance, smoking or drinking, cooking energy type, and heating energy type);

Model 4: Model 3 + insurance coverages;
Model 5: Model 4 + characteristics of the household head (marriage, education, sex, and age);
Model 6: Model 5 + housing characteristics (building type, rent, in-house telephone, in-house internet, and household tidiness).
