# Supplementary material for: Clean air actions in China, PM2.5 exposure, and household medical expenditures: A quasi-experimental study
Source: PLoS Med. 2021 Jan 6;18(1):e1003480. doi: 10.1371/journal.pmed.1003480 (PMC7787388; doi:10.1371/journal.pmed.1003480)
Supplement: S4 Table — (DOCX) [file pmed.1003480.s005.docx]

S4 Table Results of causal mediation models adjusted by different sets of covariates.

| Model^*^ | Expenditure per 10 μg/m^3^ increment in PM_2.5_ | | | Mediated proportion (%) |
| --- | --- | --- | --- | --- |
|  | Effect mediated by hospital admission | Direct effect | Total effect |  |
| 1 | 43.7 (15.3, 80.8) | 248.6 (58.3, 512.0) | 292.2 (89.5, 562.8) | 15.2 (5.3, 44.1) |
| 2 | 42.6 (11.6, 74.5) | 231.0 (56.6, 454.4) | 273.6 (83.8, 497.4) | 15.4 (6.0, 41.9) |
| 3 | 41.2 (12.1, 77.4) | 210.2 (-1.8, 382.0) | 251.4 (33.6, 427.8) | 15.6 (4.2, 57.0) |
| 4 | 35.2 (3.7, 56.1) | 185.0 (-16.7, 353.9) | 220.1 (30.3, 392.6) | 14.0 (3.6, 606.6) |
| 5 | 37.1 (4.0, 69.0) | 212.0 (85.7, 391.5) | 249.1 (109.5, 431.1) | 14.6 (1.8, 36.4) |
| 6 | 37.8 (7.1, 80.2) | 177.6 (-29.3, 387.2) | 215.4 (15.9, 410.5) | 17.2 (1.0, 150.3) |

* Model 1: Unadjusted model;
Model 2: Model 1 + nonlinear effect of temperature;
Model 3: Model 2 + household characteristics (residence, child-rearing, parental care, number of member(s) who eat together, and per capita wage) + indoor risk factors (indoor temperature maintenance, smoking or drinking, cooking energy type, and heating energy type);

Model 4: Model 3 + household characteristics (residence, child-rearing, parental care, number of member(s) who eat together, and per capita wage) + indoor risk factors (indoor temperature maintenance, smoking or drinking, cooking energy type, and heating energy type);
Model 5: Model 4 + insurance coverages;
Model 6: Model 5 + housing characteristics (building type, rent, in-house telephone, in-house internet, and household tidiness).
