# Supplementary material for: Clean air actions in China, PM2.5 exposure, and household medical expenditures: A quasi-experimental study
Source: PLoS Med. 2021 Jan 6;18(1):e1003480. doi: 10.1371/journal.pmed.1003480 (PMC7787388; doi:10.1371/journal.pmed.1003480)
Supplement: S1 Fig — In each pixel, the trend is quantified using a least-squared regression of monthly PM2.5 concentrations against the temporal index. The geographic maps were obtained from Natural Earth (http://www.naturalearthdata.com/). The places without available data (e.g., the South China sea islands) are not displayed in the maps. PM, particulate matter. (DOCX) [file pmed.1003480.s006.docx]

S1 Fig Spatiotemporal trends in PM_2.5_ before (2011-2013) and during the clean air actions (2013-2015). In each pixel, the trend is quantified using a least-squared regression of monthly PM_2.5_ concentrations against the temporal index. The geographic maps were obtained from Natural Earth (http://www.naturalearthdata.com/).
