# Supplementary material for: Clean air actions in China, PM2.5 exposure, and household medical expenditures: A quasi-experimental study
Source: PLoS Med. 2021 Jan 6;18(1):e1003480. doi: 10.1371/journal.pmed.1003480 (PMC7787388; doi:10.1371/journal.pmed.1003480)
Supplement: S2 Fig — (DOCX) [file pmed.1003480.s007.docx]

S2 Fig Results from the bootstrap simulations for errors attributable to the exposure misalignments, due to the usage of city-level PM_2.5_.
