# Supplementary material for: Clean air actions in China, PM2.5 exposure, and household medical expenditures: A quasi-experimental study
Source: PLoS Med. 2021 Jan 6;18(1):e1003480. doi: 10.1371/journal.pmed.1003480 (PMC7787388; doi:10.1371/journal.pmed.1003480)
Supplement: S1 Text — (DOCX) [file pmed.1003480.s008.docx]

**S1 Text *A bootstrap method to assess exposure misalignment errors***

For the purpose to protect confidentiality, CHARLS only published the geographic information for the surveyed households in city level. The lack of addresses resulted in an areal level assessment of PM_2.5_ exposure, which might cause misalignment error. Since the city-level averages could underestimated the variation in long-term PM_2.5_ exposures between households, briefly speaking, the misalignment error would result in an underestimated uncertainty embedded in association between health outcome and exposure. To assess measurement errors in exposure, Szpiro et al. derived a bootstrap method [20], which can be also utilized here after a simple modification. The bootstrap method can specified as follows:

1. Estimate the association (*β*) between PM_2.5_ and medical expenditures using the fully-adjusted mixed effect model (Model 6);
2. Randomly select a location within the corresponding city as the address for each household;
3. Assign *pseudo*-*true* exposure values (PM_2.5_^*^) based on the addresses and the gridded monthly maps of PM_2.5_;
4. Simulate *pseudo*-*true* outcomes (*y*^*^) based on Model 6 and the *pseudo*-*true* exposures (PM_2.5_^*^);
5. Re-estimate the association (*β*^*^) between *pseudo*-*true* outcomes (*y*^*^) and city-level exposures (PM_2.5_);
6. Repeat the steps 2-5 iteratively.

The steps 2-5 mimic the procedure of exposure misalignment and thus between-iteration difference in *β*^*^s present the effect’s variance (or uncertainty) attributable to the misalignment (Fig S2). Therefore, we used SD(*β*^*^) to quantify the uncertainty embedded in the exposure misalignment error, and used SE(*β*) (*i.e.*, the standard error estimated by model 6) to present the uncertainty attributable to model estimation. The total uncertainty could be calculated as SD = sqrt[SD^2^(*β*^*^) + SE^2^(*β*)], which could be further utilized to derive a conservative confidence interval for the estimated effect of PM_2.5_ as (*β – 1.96SD, β + 1.96SD*). Accordingly, the conservative confidence interval for our estimate on the association between PM_2.5_ and household medical expenditures is (25.3, 477.8). The result suggests after considering the misalignment error embedded in city-level exposure, the estimated effect of PM_2.5_ remains as significantly positive.
